# Global phosphoproteomic analysis identifies SRMS-regulated secondary signaling intermediates

**DOI:** 10.1186/s12953-018-0143-7

**Published:** 2018-08-18

**Authors:** Raghuveera Kumar Goel, Mona Meyer, Marta Paczkowska, Jüri Reimand, Frederick Vizeacoumar, Franco Vizeacoumar, TuKiet T. Lam, Kiven Erique Lukong

**Affiliations:** 1 Department of Biochemistry, College of Medicine, University of Saskatchewan, 107 Wiggins Road, Saskatoon, SK S7N 5E5 Canada; 2 Computational Biology Program, Ontario Institute for Cancer Research, 661 University Ave Suite 510, Toronto, ON M5G 0A3 Canada; 3 Department of Medical Biophysics, University of Toronto, 101 College Street Suite 15-701, Toronto, ON M5G 1L7 Canada; 4 Department of Pathology, Cancer Cluster, College of Medicine, University of Saskatchewan, Saskatoon, SK S7N 5E5 Canada; 5 Saskatchewan Cancer Agency, University of Saskatchewan, 107 Wiggins Road, Saskatoon, SK S7N 5E5 Canada; 6 Department of Molecular Biophysics and Biochemistry and MS & Proteomics Resource, WM Keck Foundation Biotechnology Resource Laboratory, Yale University, New Haven, CT USA

**Keywords:** SRMS, PTK70, Src, BRK, FRK, PTK6, PTK5, Non-receptor tyrosine kinase, Phosphoproteomics, Mass spectrometry

## Abstract

**Background:**

The non-receptor tyrosine kinase, SRMS (Src-related kinase lacking C-terminal regulatory tyrosine and N-terminal myristoylation sites) is a member of the BRK family kinases (BFKs) which represents an evolutionarily conserved relative of the Src family kinases (SFKs). Tyrosine kinases are known to regulate a number of cellular processes and pathways via phosphorylating substrate proteins directly and/or by partaking in signaling cross-talks leading to the indirect modulation of various signaling intermediates. In a previous study, we profiled the tyrosine-phosphoproteome of SRMS and identified multiple candidate substrates of the kinase. The broader cellular signaling intermediates of SRMS are unknown.

**Methods:**

In order to uncover the broader SRMS-regulated phosphoproteome and identify the SRMS-regulated indirect signaling intermediates, we performed label-free global phosphoproteomics analysis on cells expressing wild-type SRMS. Using computational database searching and bioinformatics analyses we characterized the dataset.

**Results:**

Our analyses identified 60 hyperphosphorylated (phosphoserine/phosphothreonine) proteins mapped from 140 hyperphosphorylated peptides. Bioinfomatics analyses identified a number of significantly enriched biological and cellular processes among which DNA repair pathways were found to be upregulated while apoptotic pathways were found to be downregulated. Analyses of motifs derived from the upregulated phosphosites identified Casein kinase 2 alpha (CK2α) as one of the major potential kinases contributing to the SRMS-dependent indirect regulation of signaling intermediates.

**Conclusions:**

Overall, our phosphoproteomics analyses identified serine/threonine phosphorylation dynamics as important secondary events of the SRMS-regulated phosphoproteome with implications in the regulation of cellular and biological processes.

**Electronic supplementary material:**

The online version of this article (10.1186/s12953-018-0143-7) contains supplementary material, which is available to authorized users.

## Background

SRMS also known as PTK70 belongs to the BRK family kinases whose other two members include Breast tumor kinase (BRK/PTK6) and Fyn-related kinase (FRK/PTK5) [1–4]. Like members of the Src family kinases as well as BRK and FRK, SRMS possesses the intermolecular-binding Src-homology 3 (SH3) and Src-homology 2 (SH2) domains and a catalytic kinase domain [5]. We previously characterized the enzymatic activity of SRMS and found that the unique 50 amino acid-long N-terminal region in SRMS regulates its enzymatic activity [5]. We further characterized Dok1 as the first substrate of SRMS [5]. Various cellular substrates and signaling intermediates have been reported for BRK and FRK which have aided the understanding of the pleiotropic roles played by these kinases [1, 2, 6]. However, unlike BRK and FRK, the cellular roles of SRMS are not well investigated. Previous studies have reported Dok1 and BRK as SRMS substrates [5, 7]. More recently, we profiled the tyrosine phosphoproteome of SRMS-expressing cells via quantitative mass spectrometry analysis and identified several candidate substrates of the kinase [8]. We also validated Vimentin and Sam68 as novel substrates of SRMS [8]. Functional gene enrichment analysis of the candidate SRMS substrates revealed that these proteins were implicated in various cellular processes such as cell growth, RNA processing and protein ubiquitination, among others [8].

Kinase-substrate interactions are known to modulate the formation of secondary and tertiary protein complexes in cells [9–11]. Such protein complexes are implicated in the regulation of other kinases thereby potentiating a concerted mode of signaling, leading to the modulation of various cellular and biological functions [12, 13]. Importantly, serine/threonine kinases constitute the majority of the mammalian kinome and evidence suggests that serine/threonine and tyrosine kinases are interconnected by several protein-protein interactions and cross-phosphorylation events, indicative of global signaling cross-talks [14–16]. This inevitably suggests that a tyrosine kinase, for instance, may likely have an impact on the greater cellular phosphoproteome, extending beyond the direct phosphorylation of its substrates. The identification of such secondary or effector phosphorylation events on signaling intermediaries would be essential towards understanding the broader mechanisms of action of kinases.

Mass spectrometry has emerged as a powerful technique to identify and quantify proteins as well as associated post-translational modifications (PTMs) in almost any biological cell/tissue type [17, 18]. Label-free quantitation techniques offer a straightforward and cost-effective approach to reliably quantify peptide and proteins abundances in cells [19–21]. The technique has gained popularity in recent years and successfully applied to several phosphoproteomics studies [22–25].

In the present study, using label-free quantitation, we investigated the SRMS-regulated global phosphoproteome to identify potential secondary phosphorylation events involving phosphoserine and phosphothreonine sites. We used the TiO_2_-based phosphopeptide enrichment strategy as a tool to preferentially identify serine/threonine phosphorylation events in cells expressing wild-type SRMS. Our analyses identified multiple significantly upregulated phosphosites in the SRMS phosphoproteome. Using bioinformatics analyses, the upregulated proteins and phosphosites were mapped to various cellular and biological processes and cognate serine/threonine kinases, respectively. Overall, our findings provide important insights on the cross-talks between SRMS and serine/threonine kinases in the orchestrated modulation of SRMS-regulated cellular functions.

## Methods

### Cell culture, plasmids and transfection

Human embryonic kidney (HEK) 293 cells were cultured at 37 °C in DMEM high glucose media (SH30243.01, Hyclone) supplemented with 10% FBS. The plasmid encoding GFP-SRMS wild-type has been previously described [5]. All transfections were performed on cells cultured to 70–80% confluency in 10 cm dishes. Briefly, 10 μg of the appropriate plasmid was first added to 430 μL of 0.15 M NaCl and gently vortexed for 10 s to mix. Next, 60 μL of 1% PEI (Polyethyleneimine, cat. #23966, Polysciences Inc.) was added to the DNA mix and vortexed briefly again. This mix was incubated at room temperature for 10mins and thereafter dispensed dropwise throughout the culture dish. The culture dishes were swirled to allow even distribution of the DNA-PEI mix and incubated at 37 °C overnight. The following day (24-h post-transfection) transfection efficiencies were verified by fluorescence microscopy.

### Antibodies

Antibodies against GFP (sc-101,525) and SRMS (sc-374,324) were procured from Santa Cruz Biotechnologies (SCBT, USA). Phosphotyrosine antibodies, 4G10 (#05–321) were purchased from EMD-Millipore (EMD-Millipore, USA). Secondary goat anti-mouse antibodies (IR Dye-800CW IgG, #926–32,210) were purchased from Li-Cor Odyssey, USA.

### Protein digestion and peptide purification

Cells expressing the GFP-tagged SRMS variants were verified under a fluorescent microscope to ensure that equivalent and over 80–90% transfection efficiencies were achieved. The transfected cells were trypsinized, washed with 1X PBS and counted. 3 × 10^6^ cells, from each condition, were lysed in RIPA buffer containing protease and phosphatase inhibitor cocktails (Pierce™, USA). For complete lysis, the cells were sonicated using three bursts of 10% amplitude followed by two bursts of 15% amplitude for 10 s each. The lysates were centrifuged at 12,000 r.c.f for 10 mins and the clarified lysates collected. Total proteins were purified by chloroform:methanol:water precipitation and the precipitated proteins resuspended in 8 M urea pH 8.0 containing 400 mM ABC. The proteins were reduced with 10 mM DTT and alkylated with 40 mM iodoacetamide followed by digestion at 37 °C with Lys-C (1:100 enzyme to protein ratio) for 6 h and trypsin (1:100 enzyme to protein ratio) overnight. The digestion reactions were quenched with 0.1% formic acid and desalted using C_18_ MacroSpin columns (The Nest Group, USA). The desalted samples were dried in a speedvac and dissolved in a buffer comprising 3.5% formic acid and 0.1% trifluoroacetic acid (TFA).

### Phosphopeptide enrichment

The samples were subjected to TiO_2_-based phosphopeptide enrichment using TopTip MicroSpin columns (Cat. #TT1TIO, Glygen Corp., USA), as described previously [26, 27]. Briefly, the samples were first acidified with 0.5% TFA and 50% acetonitrile and loaded onto the TopTip columns. The flowthrough from each sample was collected, dried in a speedvac and resuspended in 0.1% TFA for LC-MS analysis. The columns were then washed first with 100% acetonitrile, then with 0.2 M Sodium phosphate pH 7.0, 0.5% TFA, and finally with 50% acetonitrile. The enriched phosphopeptides were eluted with 28% Ammonium hydroxide, dried in a speedvac and resuspended in 0.1% TFA. Peptide concentrations in the phosphopeptide-enriched and flowthrough samples were estimated from A_280_ absorbance using the NanoDrop 2000 instrument (ThermoFisher Scientific, USA). The peptide concentrations were adjusted to 0.05 μg/μL with 0.1% TFA. A 1:10 dilution of Pierce Retention Time Calibration Mixture (Cat. #88321, ThermoFisher Scientific, USA) was further added to each sample prior to LC-MS/MS analyses.

### Mass spectrometry analyses

All samples were analysed on the Q-Exactive Plus mass spectrometer (ThermoFisher Scientific) connected to a waters nanoACQUITY UPLC system equipped with a Waters Symmetry® C18 180 μM × 20 mm trap column and a 1.7 μm, 75 μm × 250 mm nanoACQUITY UPLC column. A total of three technical replicates for each sample were analysed by LC-MS/MS. 5 μL of each replicate sample (0.05 μg/μL) was injected in a randomized order with control samples interspersed throughout to allow for correction due to potential batch effects. Peptide trapping was carried out for 3 min at 5 μL/min in 99% Buffer A (0.1% formic acid in water) and 1% Buffer B [(0.075% formic acid in acetonitrile] prior to eluting with linear gradients that reached 30% Buffer B at 140 min, 40% Buffer B at 155 min, and 85% Buffer B at 160 min. Two blanks (1st blank comprising 100% acetonitrile and 2nd blank comprising Buffer A) followed each injection to ensure against sample carry over. The mass spectrometer was operated in the Data-Dependent mode with the Orbitrap operating at 60,000 FWHM and 17,500 FWHM for MS and MS/MS, respectively. Full scans were acquired at a resolution of 60,000 FWHM with a maximum injection time of 120 ms in the Orbitrap analyzer. The fifteen most abundant ions, with charge states ≥2, were selected for fragmentation by HCD (MS/MS) using normalized collision energy set to 28 and analyzed at a resolution of 17,500 FWHM with a maximum injection time of 60 ms.

### Data processing and analyses

Label-free quantitation of the raw LC-MS/MS data was performed using the Progenesis QI software (Version 3.0, Nonlinear Dynamics), as described before [27, 28]. All MS/MS spectra were searched against the SWISSProt database (taxonomy restricted to *Homo sapiens*, database version dated November 2015) using the Mascot search engine (Version 2.0). Data from the TiO_2_-enriched and flow through fractions were analyzed separately, and the resulting quantitative analyses were combined for the respective experimental conditions. Chromatographic/spectral alignment, mass spectral peak-picking, data filtering and statistical analyses for the protein and peptide quantitation was performed on Progenesis QI. Raw mass spectral features were aligned based on their retention time using a randomly selected reference run. All other runs were automatically aligned to the reference run to minimize retention time variability between the runs. Only spectra with ion signals quantified at or above three times the standard deviation of the noise, were selected for subsequent analyses. A normalization factor for each run was calculated to account for the variability in sample loading and ionization. This normalization factor was determined by calculating the quantitative ratio of the reference run to the run being normalized, with the assumption that most proteins/peptides are not changing in the experiment. The experimental set-up grouped replicates of each condition for comparative analyses. The algorithm then calculated and tabulated raw and normalized abundances and ANOVA *p*-values (calculated as the mean difference and variance associated with the replicate data in both experimental conditions) for each quantified peptide in the dataset. The MS/MS spectra was exported as a .mgf file (Mascot generic files) for database searching. The Mascot search algorithm was used for database searching. Carbamidomethylation (Cysteine), phosphorylation (Serine, Threonine, Tyrosine), deamidation (Asparagine, Glutamine), acetylation (Lysine) and oxidation (Methionine) were specified as variable modifications. Two missed tryptic cleavages were allowed. The precursor mass tolerance was set to 10 ppm and the fragment mass tolerance was set to 0.2 Da. Stringent conditions were set in Mascot to filter out low scoring peptides by imposing a confidence probability score (*p*) of < 0.05. Peptide and protein identifications were filtered at 1% FDR using the target-decoy strategy [29]. Only phosphopeptides mapping to proteins with at least 2 tryptic peptides were considered for analyses. The Mascot search results was exported as .xml files and then imported into the processed dataset in Progenesis QI software where the Mascot-generated peptide identifications were assigned to the corresponding quantified features. Scatterplots illustrating Pearson’s correlation coefficients and fold-change phosphopeptide abundance were generated in R using in-house scripts.

### Motif enrichment analyses

Phosphopeptide sequences of 13 amino acids in length that were significantly altered between the experimental conditions (*p*-value = 0.05 and 3-fold upregulation) were searched for consensus sequences using the Motif-x [30] and PHOSIDA [31] algorithms. On Motif-x, the minimum number of occurrences for a motif in the dataset and the required motif significance was set to 20 and 10E-06, respectively. On PHOSIDA, the minimum score for phospho-Ser/Thr/Tyr was specified as 10 and the minimum proportion of matching sites was set to 5%.

### IPA functional enrichment analysis

Genes mapped from significantly upregulated phosphopeptides were used for identifying cellular and molecular processes, pathways and upstream regulators using the Ingenuity pathway analysis (IPA) software (QIAGEN Redwood City, http://www.qiagen.com/ingenuity). Genes were queried against the Ingenuity knowledge database as the reference set. The analysis was restricted to the documentation of experimentally observed findings on human genes. The Benjamini-Hochberg (B-H) multiple testing correction (B-H corrected *p*-value < 0.05) scoring method was used to compute the significance for the functional enrichment analysis. Upstream regulators refer to the upstream proteins that are responsible for causing changes in the phosphorylation and/or total expression levels of the queried genes/proteins in the dataset. Activation z-scores were calculated by IPA’s z-score algorithm to predict the overall activation or inhibition of the identified functional cellular processes/pathways and upstream regulators. A positive z-score (z-score > 0) implies an overall predicted activation of the process/pathway/upstream regulator whereas a negative z-score (z-score < 0) implies an overall predicted inhibition or downregulation of the pathway/process/upstream regulator. z-scores ≥2 or ≤ − 2 are considered significant by IPA for predicted activation or inhibition, respectively. Cellular processes/upstream regulators with no z-scores imply that IPA was unable to generate prediction states for these functionalities.

## Results

### Global analysis of the SRMS-regulated phosphoproteome

**Fig. 1:**
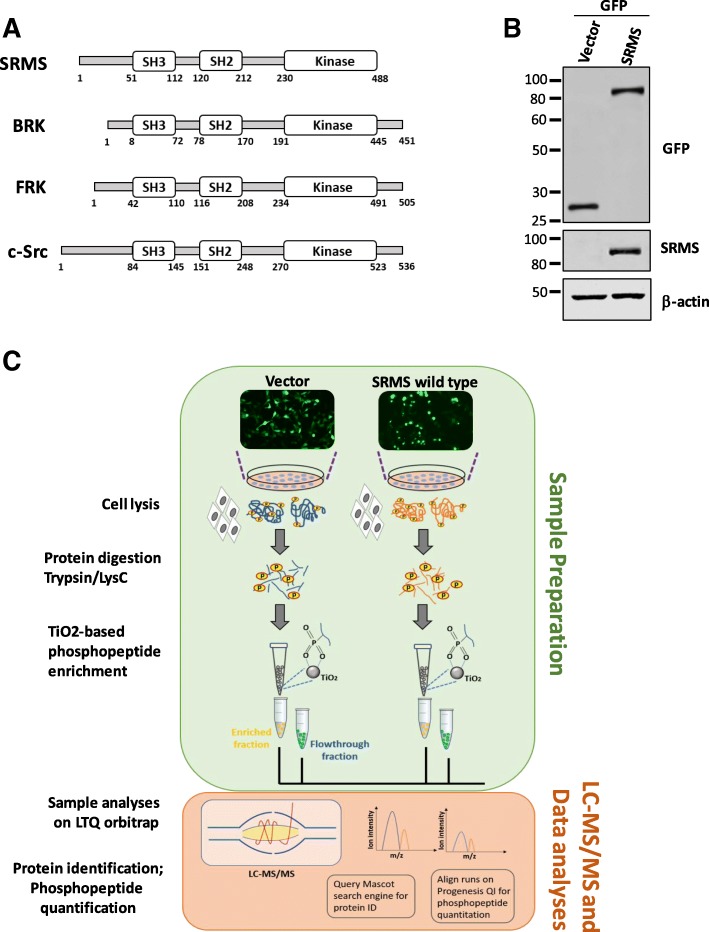
Phosphoproteomics analyses of cells expressing ectopic wild-type SRMS. **a** Schematic representation of the domain structure of SRMS, BRK and FRK (BRK family kinases), and c-Src, depicting the SH3, SH2 and kinase domains. The amino acid numbers indicate the length of the domains and the full-length protein. **b** Immunoblotting analyses was performed on a portion of the lysates derived from HEK293 cells expressing either the empty vector (GFP alone) or vector expressing GFP-SRMS (wild-type) and used for subsequent phosphopeptide enrichment analysis. The lysates were probed with antibodies against GFP and SRMS. Immunoblotting with antibodies against β-actin was used to assess the loading of total proteins. **c** Schematic representation of the label-free quantitation-based phosphoproteomics workflow using cells expressing GFP alone (the empty vector control) or cells expressing GFP-SRMS wild type. The cells were lysed in RIPA buffer followed by dual enzymatic digestion (Trypsin/Lys-C) and phosphopeptide enrichment using TiO_2_ resin. Both, enriched and flowthrough fractions were analysed by LC-MS/MS and data analyses performed using the MASCOT search engine (for protein identification) and PROGENESIS QI tool (for phosphopeptide quantitation)

Tyrosine kinases and serine/threonine kinases are known to exhibit dynamic cross-talks leading to a concerted mode of regulation of signaling networks [14–16]. Evidence from large-scale database curation and high-throughput experimental observations indicate that such cross-talks are modulated through secondary and tertiary protein-protein interactions around primary kinase-substrate interaction and phosphorylation events [12, 14]. We therefore sought to study the SRMS-regulated serine/threonine phosphoproteome to infer cues on its contribution to the cellular signaling network potentially regulated by SRMS. To survey these global phosphoproteomic changes we performed metal-ion enrichment-based quantitative phosphoproteomics analysis on cells ectopically expressing wild-type SRMS (Fig. 1a). Cells expressing the empty vector backbone served as control for all background phosphorylation events occurring natively in HEK293 cells. We confirmed the expression of wild-type SRMS in the cells by immunoblotting the lysates with antibodies against GFP and SRMS (Fig. 1b). As expected, immunoblotting with phosphotyrosine antibodies confirmed the enzymatic activation of wild-type SRMS in these cells (Additional file 1: Figure S1). The total proteins were then subjected dual enzymatic digestion with Lys-C and trypsin followed by phosphopeptide enrichment using titanium dioxide (TiO_2_) resin, prior to LC-MS/MS analyses (Fig. 1c).

**Fig. 2:**
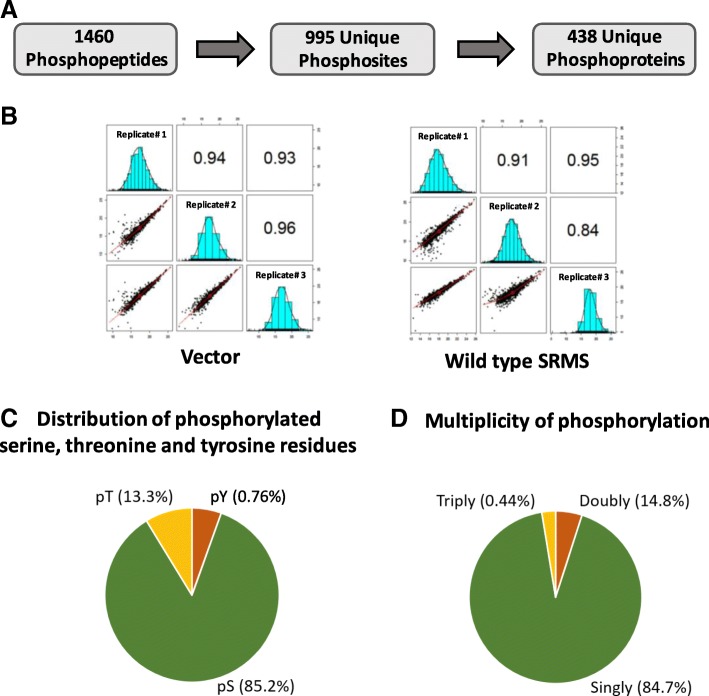
Identification of the phosphoproteome. **a** The coverage of the phosphoproteome showing the total number of identified phosphopeptides, unique phosphosites and unique proteins mapping from the phosphopeptides. **b** Pearson’s correlation analyses of the 3 replicate datasets corresponding to the vector control and wild type SRMS phosphoproteome. The Pearson’s correlation coefficients and associated distribution curve histograms between Replicate 1, 2 and 3 for both experimental conditions are reflected in a matrix format. **c** Pie-chart representation of the multiplicity of phosphorylation of the identified phosphopeptides indicating the percentage of phosphopeptides carrying either a single phosphosite, double phosphosites or triple phosphosites. **d** Pie-chart depiction of the proportion of phosphoserine, phosphothreonine and phosphotyrosine sites in the identified phosphoproteome

The experiment was performed in three replicates to ensure statistical reproducibility of our analysis. Collectively, we identified 995 unique phosphosites from 1459 redundant phosphopeptides which mapped to 439 unique phosphoproteins, at an estimated False Discovery Rate (FDR) of 1% (Fig. 2a, Additional file 2: Table S1). We found significant reproducibility between the replicate datasets, as reflected by a Pearson’s correlation coefficient in the range of 0.84 to 0.96 (Fig. 2b). We quantified the number of phosphoserine, phosphothreonine and phosphotyrosine sites to assess the distribution of the identified phosphoproteome. Consistent with previous studies employing metal ion-based phosphopeptide enrichment techniques [32–34], we observed that the majority of the identified phosphosites were represented by phosphoserine (85.2%) followed by phosphothreonine sites (13.3%) (Fig. 2c). Phosphotyrosine sites represented a minor fraction (0.76%) of the identified phosphoproteome (Fig. 2c). This was an expected observation since previous studies have reported a similar phosphotyrosine enrichment profile using metal ion enrichment chromatography despite significant induction of cellular tyrosine phosphorylation, for instance, via pervanadate treatment [34, 35]. Additionally, singly phosphorylated peptides were more strongly represented than doubly or triply phosphorylated peptides in our dataset, consistent with previous reports [33, 36, 37] (Fig. 2d).

**Fig. 3:**
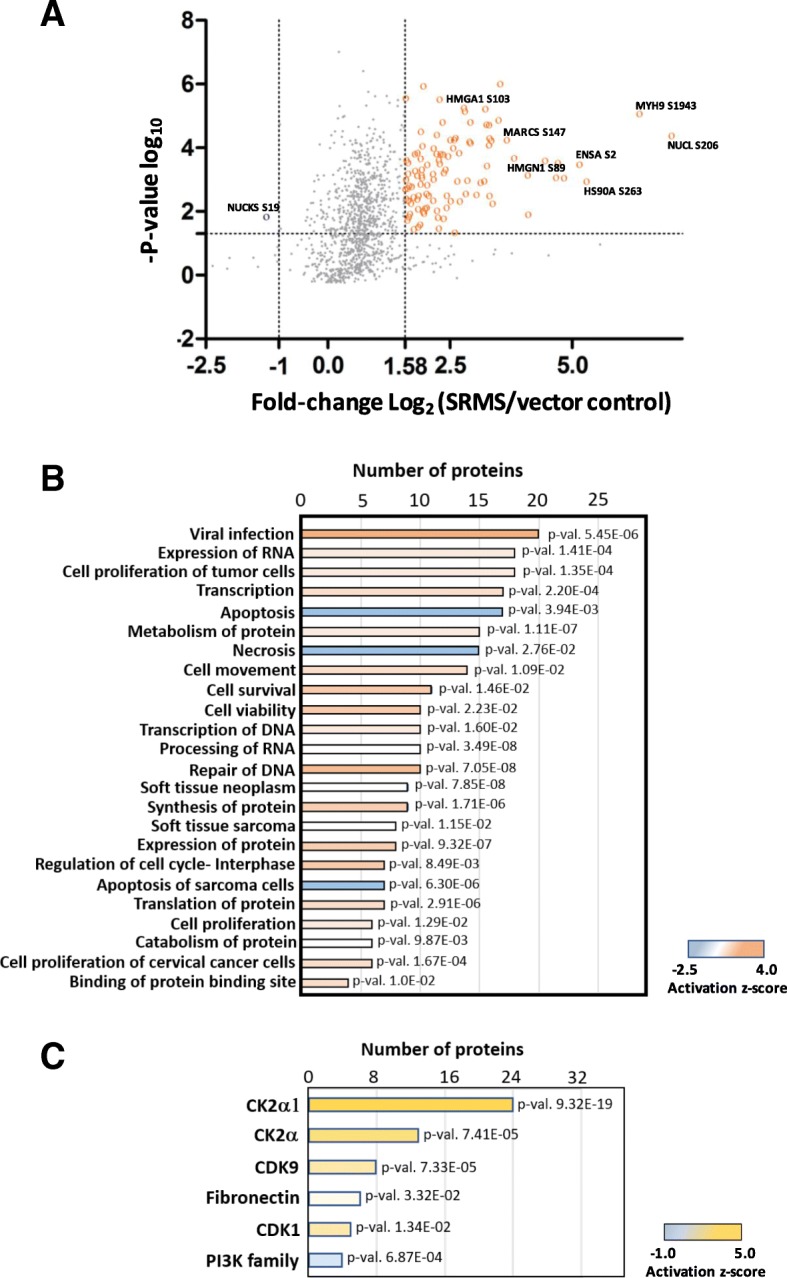
Functional enrichment analyses of the significantly altered phosphoproteins. **a** Scatter plot showing the phosphopeptide log_2_ fold-change (SRMS/control) plotted against the -Log_10_ *p*-value highlighting the significantly regulated phosphopeptides (ANOVA *p*-value ≤0.05, upregulation fold-change cut-off = Log_2_ ≥ 1.58 and downregulation fold-change cut-off = Log_2_ ≤ − 1). Upregulated phosphopeptides are highlighted in orange while downregulated phosphopeptides are highlighted in blue. **b** IPA analyses of the top cellular and molecular processes enriched from upregulated phosphoproteins identified in the SRMS-regulated phosphoproteome (*n* = 60; corresponding to upregulated phosphopeptides; SRMS/Control Log_2_ ≥ 1.58-fold). The activation z-score indicates the predicted upregulation (z-score > 1) or downregulation (z-score < 0) of specific cellular and molecular processes. **c** IPA analyses of upstream regulators for the upregulated phosphoproteins (*n* =  60). z-score indicates the predicted activation (z-score > 1) or inactivation (z-score < 0) of the indicated upstream proteins

To gain a better understanding of the SRMS-regulated phosphoproteome, we first applied a *p*-value threshold of ≤0.05 to focus on phosphopeptides that displayed statistically significant differential regulation (up or downregulation) compared to control cells (Fig. 3a and Additional file 2: Table S1). We then defined significantly regulated phosphopeptides in the wild-type SRMS phosphoproteome as a measure of the differential abundance of the corresponding average pre-cursor ion intensities in the control cells. To ensure stringent analyses, phosphopeptides quantified at average pre-cursor ion intensities ≥3-fold [Log_2_ (SRMS/control) ≥ 1.584] compared to corresponding average intensities in control cells were considered hyperphosphorylated or upregulated (Fig. 3a). Similarly, phosphopeptides with average intensities ≤0.5-fold [Log_2_ (SRMS/control) ≤ − 1; conversely equal to 2-fold upregulation in control cells] were considered hypophosphorylated or downregulated (Fig. 3a). Using these criteria, we identified 140 upregulated and 1 downregulated (NUCKS S19) phosphopeptides in the wild-type SRMS-regulated phosphoproteome (Fig. 3a and Additional file 2: Table S1). Some of the significantly upregulated phosphosites included NUCL S206, NUCL S184, MYH9 S1943, HS90A S263 and MARCS S147. Overall, these phosphopeptides mapped to 60 upregulated and 1 downregulated proteins, respectively (Table 1 and Additional file 2: Table S1). (Table 1 is appended after the “Discussions” section in the present manuscript). We used the 0.5-fold cut-off for downregulation since our dataset comprised only 37 phosphopeptides below the median 1-fold differential abundance (*p*-value ≤0.05) and most of these peptides displayed only marginal downregulation in phosphorylation (SRMS/Control ~ 0.7-fold). Additionally, we reasoned that phosphopeptides that are at least 2-fold more abundant in control than in the SRMS-regulated phosphoproteome would be likely to represent genuine SRMS-regulated hypophosphorylation events.

**Table 1:** SRMS-dependent upregulated phosphoproteins

Protein Accession (UniProt ID)	Phosphosite(s)	Protein Description
AHNK	S5731, S5739	Neuroblast differentiation- associated protein
AKA12	S248, S381, S732, S743, S749	A-kinase anchor protein 12
BAD	S75, S118	Bcl-associated agonist of cell death
CALX	S554, S564	Calnexin
DKC1	S451, S453, S455, S458, S494	H/ACA ribonucleoprotein complex subunit 4
FOXO3	T51, S55	Forkhead box protein O3
HS90	S252, S263	Heat shock protein HSP 90-alpha
HMGA1	S102	High mobility group protein HMG-I/HMG-Y
IF2P	S107, S113	Eukaryotic translation initiation factor 5B
MAP1B	S1016, S1154, S1869, T1879	Microtubule-associated protein 1B
MARCS	S147, T150	Myristoylated alanine-rich C-kinase substrate
NUCL	S145, S184, S206	Nucleolin
NUDC	S136, S139, S145	Nuclear migration protein nudC
SFR19	Y305, T335, T994, S998, T1001	Splicing factor, argining/serine-rich 19
SRRM1	S465, S775	Serine/arginine repetitive matrix protein 1
SRRM2	S144, T1413	Serine/arginine repetitive matrix protein 2
TCP4	S9, S17, S19	Activated RNA polymerase II transcriptional coactivator p15

Shown here is a representative list of selected hyperphosphorylated/upregulated proteins identified in the wild-type SRMS phosphoproteome

### Functional annotation of the SRMS-regulated phosphoproteome

To better understand the cellular and biological processes mapped from the SRMS-regulated phosphoproteins, we performed functional gene enrichment analyses using the Ingenuity Pathway analyses (IPA) tool. We increased the stringency of our analyses by restricting functional annotations inferred exclusively from experimental observations. This led us to identify 30 molecular and cellular processes (Benjamini-Hochberg multiple testing *p*-value < 0.05) mapped from the majority of the hyperphosphorylated proteins (48/60 proteins or 80%) identified in the SRMS phosphoproteome (Fig. 3b and Additional file 3: Table S2). These cellular processes broadly represented key functional categories that included protein synthesis (*p*-value range 1.11E-07 to 9.87E-03), cell cycle (*p*-value range 8.49E-03 to 1.03E-02), RNA post-transcriptional modification (*p*-value range 3.49E-08 to 1.95E-04), cell death and survival (*p*-value range 6.30E-06 to 2.76E-02), cell growth and proliferation (*p*-value range 1.35E-04 to 1.29E-02) and DNA replication and repair (*p*-value 7.05E-08 and 1.02E-04). Importantly, our analyses also led us to determine the predicted activation states of 4 molecular and cellular processes at a significant activation z-score threshold of ±2. Specifically, cellular processes related to viral infection (20 proteins, z-score = 3.8, *p*-value = 5.45E-06) and DNA repair (10 proteins, z-score = 2.18, *p*-value = 7.05E-08) were predicted with a significantly increased activation state while apoptosis (17 proteins, z-score = − 2.11, *p*-value = 3.94E-03) and necrosis (15 proteins, z-score = − 2.48, *p*-value = 2.76E-02) were predicted to display an overall decreased activation state (Fig. 3b and Additional file 3: Table S2). Some of the other interesting cellular/biological themes enriched from the hyperphosphorylated proteins included cell movement (*p*-value = 1.35E-04) and catabolism of protein (*p*-value = 9.87E-03) (Fig. 3b and Additional file 3: Table S2). Analyses of the upstream regulators identified 5 proteins, namely, PI3K (Phosphoinositol-3-kinase), CDK9 (Cyclin-dependent kinase 9), CK2 (Casein kinase 2), CK2α (Casein kinase 2 catalytic subunit alpha) and FN1 (Fibronectin) (Fig. 3c and Additional file 3: Table S2). Importantly, among these, 2 kinases were associated with significantly increased predicted activation- the tetrameric kinase complex, CK2 (z-score = 3.54, *p*-value = 7.41E-05), its monomeric catalytic subunit, CK2α (z-score = 4.87, *p*-value = 9.32E-19), and CDK9 (z-score = 2.81, *p*-value = 7.33E-05). Further, our analyses also revealed that a small number of the hyperphosphorylated proteins were enriched in specific signaling pathways (Additional file 3: Table S2) which included Telomerase signaling (−Log *p*-value = 2.33), PI3K/AKT signaling (−Log *p*-value = 2.18), Sirtuin signaling (−Log *p*-value = 1.93) and Phospholipase C signaling (−Log *p*-value = 1.44).

We next used the STRING database to generate a protein-interaction network of the SRMS-regulated hyperphosphorylated proteins. STRING allows for the analyses of protein-protein interactions of a given set of genes based on correlation with predicted and experimental sources [38]. Our analyses using STRING’s curated database of human protein interactions resulted in a network of 38 interconnected nodes (proteins) characterized by 59 edges (connections) implying a moderate level of interaction among the hyperphosphorylated proteins (Additional file 4: Figure S2). This reasonably moderate degree of connectivity among 38 phosphoproteins (63% of total SRMS-regulated hyperphosphorylated proteins) suggests that a sizeable number of the SRMS-regulated phosphoproteins potentially partake in the same protein complex and possibly the same cellular signaling processes.

### Analyses of phosphorylation motifs and predicted kinases

**Fig. 4:**
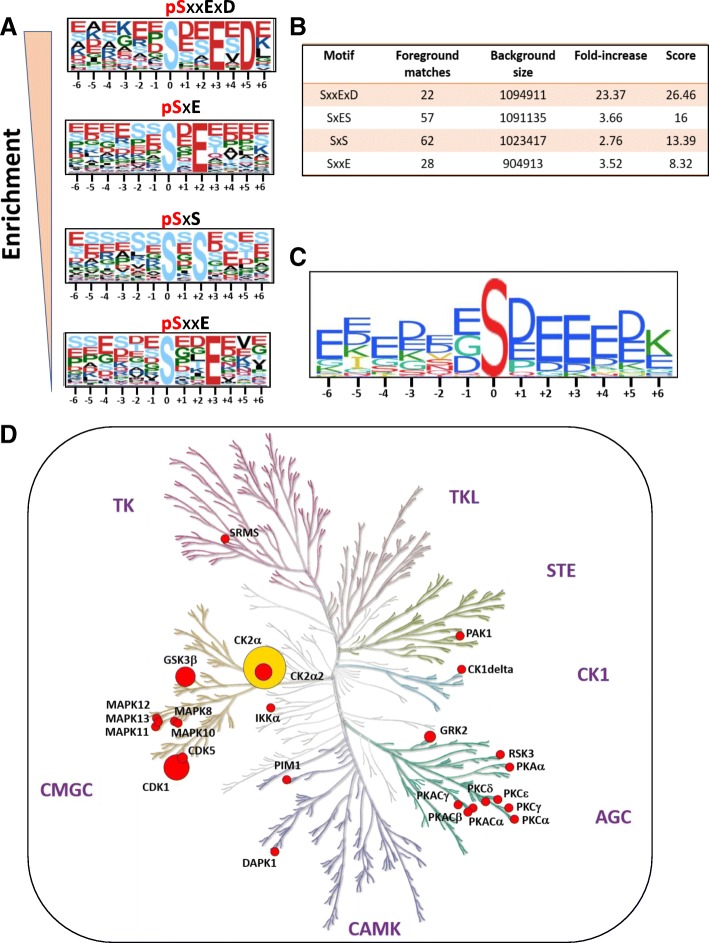
Motif-enrichment analyses of SRMS-dependent upregulated phosphopeptides. **a** Motif-logos showing the significantly enriched motifs (*p*-value <10E-06) identified by Motif-x [30]. The positions of the amino acid residues C-terminal or N-terminal to the central phosphoresidue (position “0”) are shown in the logos. The height of the amino acid residues is proportional to their enrichment at the specific position in the pool of the queried phosphopeptides. **b** Table representing the scoring information relevant to the enriched motifs identified by Motif-x. **c** A consolidated motif-logo generated by PHOSIDA [31] showing the enrichment of various amino-acid residues at specific positions relative to the central phosphoresidue (position “0”). **d** Dendogram of the human kinome, constructed by KinMap [69], highlighting the candidate kinases predicted to target the upregulated phosphopeptides, as identified by NetworKIN [42] analysis. Node size is proportional to the number of the queried phosphosites targeted by the kinase. CK2 (node highlighted in yellow) was identified as the upstream kinase for the maximum number of queried phosphosites. Major kinase families are annotated in the dendogram which include: TK (Tyrosine Kinases), TKL (Tyrosine Kinase-Like), STE (Sterile kinases; homologs of the yeast STE7, STE11 and STE20 kinases), CK1 (Casein Kinase 1), AGC (comprising Protein kinase A/ PKA, PKG and PKC kinase sub-families), CAMK (Calcium/Calmodulin-dependent kinases) and CMGC (comprising cyclin-dependent kinase (CDK), mitogen-activated protein kinase (MAPK), glycogen synthase kinase (GSK) and CDC-like kinase (CLK))

The amino acids surrounding a phosphosite constitute important recognition motifs for cognate kinases [39–41]. Our phosphoproteomics analysis identified 140 upregulated phosphopeptides that mapped to 60 proteins in the SRMS-regulated phosphoproteome. We assessed the enrichment of motifs among these upregulated phosphopeptides using the Motif-x and PHOSIDA motif-enrichment tools [30, 31]. Our analyses using both the tools resulted in the enrichment of similar motifs (*p*-value = 10E-06) which primarily corresponded to phosphoserine sites (Fig. 4a, b and c). No significant phosphothreonine or phosphotyrosine motifs were found due to their lower abundances in our dataset. Collectively, we identified four major motifs; all comprising positional variations in amino acids following the central phosphoserine residue. One significantly overrepresented motif comprised a glutamic acid and aspartic acid residue in the + 3 and + 5 positions (*SxxExD*) (Fig. 4a, b and c). Other top motifs comprised either a glutamic acid and a serine residue at the + 2 and + 3 positions (*SxES*), a serine at + 2 position (*SxS*) or a glutamic acid residue at + 3 position (*SxxE*) (Fig. 4a, b and c).

We further analyzed the upregulated phosphopeptides using the NetworKIN tool [42] to identify candidate kinases responsible for the site-specific phosphorylation of the associated motifs. The NetworKIN algorithm exploits information based on consensus motif recognition as well as context-specific factors such as the physical association and co-expression of kinases and substrates, to predict corresponding kinase-substrate relationships [42, 43]. NetworKIN analyses of the hyperphosphorylated motifs identified 25 candidate serine/threonine kinases, corresponding to 16 kinase subfamilies, at a NetworKIN score > 3 (Fig. 4d and Additional file 5: Table S3). Kinases found to potentially target multiple phosphosites included CDK1 (5 phosphosites, NetworKIN score range: 9.62–17.93), CK2 alpha (19 phosphosites, score range: 3.17–35.63), GSK3 beta (5 phosphosites, score range: 4.75–10.43), CK2 alpha 2 (3 phosphosites, score range: 8.47–9.61), GRK2 (3 phosphosites, score range: 4.30–9.76) and CDK5 (2 phosphosites, score range: 3.42–14.02) (Fig. 4d and Additional file 5: Table S3). Importantly, Casein Kinase 2 alpha (CK2α) was the among the highest scoring candidate kinases and found to target the maximum number of phosphosites in our dataset (Fig. 4d and Additional file 5: Table S3). CK2α is a functionally independent catalytic subunit of the tetrameric holoenzyme, CK2 [44]. The consensus recognition motifs for the acidophilic kinase, CK2α, are characterized by the presence of either glutamic acid or aspartic acid residues near the phosphorylation site [43, 45]. Indeed, the overrepresentation of such motifs (*SxxExD, SxE and SxxE*) by motif enrichment analyses confirms CK2α as a major upstream kinase of the upregulated phosphosites. This also corroborated findings from our functional gene enrichment analyses using IPA where the holoenzyme CK2 and its catalytic module, Casein kinase 2 alpha were identified as major upstream regulatory kinases of the upregulated phosphoproteins (Fig. 3c). Taken together, our findings suggest that CK2α represents a key serine/threonine kinase that is potentially activated in the SRMS phosphoproteome. This further indicates that CK2a and/or CK2 may likely represent key downstream targets of SRMS.

## Discussion

Here we used quantitative mass spectrometry analyses to identify cognate serine/threonine phosphorylation events significantly altered in the SRMS-regulated phosphoproteome. We identified 60 phosphoproteins mapped from 140 phosphopeptides which were upregulated by at least 3-fold in the SRMS-regulated phosphoproteome. To our knowledge, this represents the first study to investigate global serine and threonine phosphorylation changes induced by a bonafide non-receptor tyrosine kinase in the eukaryotic phosphoproteome.

Despite the ectopic expression of SRMS in cells, we identified only 1.3% phosphotyrosine peptides in contrast to our previous study where we reported multiple phosphotyrosine peptides [8] (Fig. 2d). The lysis of cells under less stringent conditions, such as by RIPA lysis buffer used in this study, and the use of TiO_2_ resin, known to less favorably bind to phosphotyrosines, are factors that may explain the lower enrichment of tyrosine-phosphorylated peptides. The former may be an equally important factor since tyrosine phosphorylation is far more stringently regulated by phosphatase activity than serine/threonine phosphorylation [22] and as such the use of denaturing conditions during cell lysis have proved useful in such studies [22, 46, 47]. The relatively lower selectivity of TiO_2_ resin towards phosphotyrosine peptides can also be demonstrated by the fact that we identified only 2 major phosphorylation sites on SRMS- Y299 and Y456, in this study. Our previous phosphotyrosine enrichment-based approach enabled the identification of several other phosphotyrosine sites on wild-type SRMS [8]. However, since the focus of this study was to identify phosphoserine and phosphothreonine events, the relatively poor enrichment of phosphotyrosine peptides did not pose a concern.

The cellular roles of SRMS have not been well characterized to date. In the present study, functional enrichment analyses of the upregulated phosphoproteins in the SRMS-regulated phosphoproteome identified apoptosis and necrosis as potentially important downregulated cellular processes in cells expressing ectopic SRMS. A previous study by Potts et al. implicated SRMS in the potential negative regulation of autophagy [48], implying that SRMS may potentially be a positive regulator of stress-induced cell survival. This is in line with our present findings which not only projected a downregulation of apoptotic processes but also a potential upregulation of cell survival and proliferative processes in cells expressing ectopic SRMS, as determined by a positive z-score from IPA analyses (Fig. 3b and Additional file 3: Table S2). Interestingly, processes related to DNA repair were also predicted to be upregulated (Fig. 3b and Additional file 3: Table S2). As an example, HMGA1 was among the upregulated proteins enriched in DNA repair processes (Additional file 3: Table S2). We identified HMGA1 S102 as a hyperphosphorylated site in cells expressing ectopic SRMS (Additional file 2: Table S1). HMGA1 S102 is a target site for phosphorylation by CK2 [49, 50] and studies have shown that the hyperphosphorylation of HMGA1 S102 impairs the DNA-binding ability of the protein [51, 52]. The dissociation of HMGA1 from the DNA in turn promotes efficient DNA repair, presumably by allowing various DNA repair factors to be recruited to the sites of DNA lesions, as reported previously [53, 54]. Taken together, our data points towards a potential role of SRMS in modulating cellular DNA repair processes by regulating HMGA1 S102 phosphorylation. Further, HMGA1 has also been shown to inhibit apoptosis by suppressing p53-mediated transcriptional repression of apoptosis-related genes like Mdm2, Bax and p21 [55]. This therefore also explains the cross-talk involving HMGA1 between cellular processes linked to DNA repair, apoptosis and ell proliferation, in our dataset (Additional file 3: Table S2).

Analyses of the phosphosites using the Motif-x, PHOSIDA and NetworKIN tools identified CK2 as one of the major candidate upstream kinase for the upregulated phosphosites identified in the SRMS-regulated phosphoproteome (Fig. 4a-d, Additional file 3: Table S2). This raises the possibility that SRMS may potentially function upstream of CK2 and as a candidate regulator of CK2 activity. CK2 is an active tetrameric serine/threonine kinase composed of two catalytic subunits (α and α’) and two regulatory β-subunits [56]. Activation of CK2 is primarily regulated in vivo by inositol phosphates [57, 58], phospholipase D2 (PLD2) and protein kinase C (PKC) [59]. However, previous studies have identified c-Abl, BCR-Abl [60] and Src-family kinases, Lyn and c-Fgr [61] as regulators of CK2 activity. Specifically, the catalytic subunit of CK2, CK2α, was identified as a substrate of these kinases where phosphorylation by c-Abl or BCR-Abl was shown to inhibit CK2α activity [60] while phosphorylation by Lyn or c-Fgr was shown to increase CK2α activity [61]. Interestingly, our previous phosphotyrosine enrichment-based phosphoproteomics analysis found CK2α to be significantly tyrosine-phosphorylated exclusively in cells expressing ectopic wild type SRMS, projecting CK2α as a candidate SRMS substrate [8]. Specifically, CK2α Y182, Y188 and Y323 were identified as hyperphosphorylated sites. Of these, Y182 represents a key residue lying in the activation loop of the CK2α subunit and the trans-autophosphorylation of this site has been shown to increase CK2α activity [62]. Additionally, CK2 Y188 has also been reported as another activation loop phosphosite contributing to CK2α activation, albeit to a lesser extent than CK2 Y182 [62, 63]. However, findings from our previous phosphoproteomics study indicate that CK2α Y182/Y188 may potentially serve as target sites of SRMS leading to the SRMS-dependent modulation of CK2α enzymatic activity. Overall, this supports our present findings which indicate that CK2α is potentially activated in cells overexpressing wild type SRMS which may highlight SRMS as a possible modulator of CK2α kinase-dependent functions.

CDK1/cdc2 is another upstream candidate kinase identified by NetworKIN analysis which was also identified as an upstream regulator by IPA analyses. Kinases Wee1 [64], Myt1 [65] and Lyn [66] are known to regulate the activity of CDK1 by phosphorylating the inhibitory Y15 site on CDK1. Our previous phosphoproteomics study also identified CDK1 as a candidate target of SRMS where CDK1 Y19, Y270 and Y286 were found to be hyperphosphorylated [8]. However, these sites on CDK1 have not been functionally characterized. Therefore, the mechanism by which CDK1 may be potentially activated in the presence of SRMS, leading to the phosphorylation of the CDK1 consensus phosphosites, is not known.

It is important to note that the overall cellular and molecular processes mapped from the SRMS-regulated signaling intermediates identified in the present study, were consistent with the major functional themes mapped from the candidate SRMS substrates in our previous study [8]. Some of the major functional themes enriched by the candidate SRMS substrates included RNA processing, Viral processes, negative regulation of apoptosis, cell cycle regulation and protein ubiquitination [8]. These correlate well with the broad functional categories identified in the present study such as RNA post-transcriptional modifications (Processing of RNA), viral infection, cell death and survival (apoptosis), cell cycle, and protein degradation, respectively (Additional file 3: Table S2). Collectively, our findings here indicate that some of the major cellular processes regulated by SRMS involve additional signaling factors characterized by serine/threonine phosphorylation events downstream of cognate serine/threonine kinases. Our study therefore presents key evidence that serine/threonine phosphorylation forms part of important secondary signaling events triggered by SRMS. Overall, our findings provide an important mechanistic resource to characterize the cellular roles played by SRMS.

## Conclusions

Our global phosphoproteomic profiling reveals that the non-receptor tyrosine kinase, SRMS can indirectly regulate multiple signaling intermediates which are characterized by an altered serine/threonine phosphorylation status. The regulation of serine/threonine phosphorylation events in the presence of SRMS is likely a result of SRMS-dependent direct or indirect modulation of serine/threonine kinase activity. In this context, the serine/threonine kinase, Casein kinase 2 (CK2) may likely represent one of the major downstream targets of SRMS. Overall, findings from our study form an important mechanistic resource for characterizing the cellular role(s) of SRMS in mammalian cells.

## Additional files

Additional file 1: **Figure S1.** Immunoblotting analysis with antibodies against total phosphotyrosines on lysates derived from cells expressing either empty vector control (GFP alone) or GFP-SRMS wild type. Antibodies against β-actin were used for immunoblotting to assess the loading of total proteins. (TIF 855 kb)

Additional file 2: **Table S1.** The table is organized in the following spreadsheets: Spreadsheet “All Phosphopeptides”: This table lists all identified phosphopeptides from three independent replicates, via LC-MS/MS. Additional information corresponding to each phosphopeptide is also shown and includes peptide ID# (numerical ID), fraction (enriched; 1 or flowthrough; 2 fractions), Retention time, m/z, charge, measured mass, mass error, score, peptide sequence, modifications (type of PTM), UniProt accession, Phosphosite(s), sequence window, description of protein, use in quantitation (at least 2 unique tryptic peptides were identified for the corresponding protein), ANOVA *p*-values corresponding to each phosphopeptide identified across the three independent replicates, normalized abundance (phosphopeptide intensity) of the identified phosphopeptides across each replicate, average phosphopeptide intensities of the peptide in the control cells or cells expressing wild type SRMS, fold-change abundance of phosphopeptide intensities in SRMS-expressing cells (SRMS/Control) and Log_2_-scaled fold-change of SRMS/control values. Spreadsheet “All unique phosphoproteins”: This table lists all the unique phosphoproteins mapped from the phosphopeptides quantified via LC-MS/MS in the Vector and SRMS-expressing cells. Also shown are all associated phosphosites identified for each protein. Spreadsheet “Phosphopeptides *p*-value ≤ 0.05”: This table lists all phosphopeptides filtered at an ANOVA *p*-value threshold of 0.05. Also shown is other relevant information corresponding to each phosphopeptide, as in Spreadsheet “All Phosphopeptides”. Spreadsheet “Hyperphosphorylated proteins”: This table lists all the hyperphosphorylated/upregulated proteins identified in the SRMS-regulated phosphoproteome. These proteins were mapped from the hyperphosphorylated/upregulated peptides (Log_2_ SRMS/Control ≥1.58 and ANOVA *p*-value ≤0.05). Therefore, only phosphosites identified at Log_2_ SRMS/Control ≥1.58 and ANOVA *p*-value ≤0.05, are shown for every protein. Spreadsheet “Hypophosphorylated proteins”: Shown here is the hypophosphorylated/downregulated protein mapped from the hypophosphorylated/downregulated peptide (Log_2_ SRMS/Control ≤ − 1 and ANOVA *p*-value ≤0.05) identified in SRMS-expressing cells. (XLSX 745 kb)

Additional file 3: **Table S2.** The file is organized into the following spreadsheets: Spreadsheet “Cell. & Biol. processes”: This table shows all the cellular and biological processes identified by IPA analysis (www.ingenuity.com) enriched from the upregulated/hyperphosphorylated proteins identified in the SRMS-regulated phosphoproteome. To minimize redundancy, the cellular and biological processes are categorized in specific functional “categories”. Also shown for every cellular and biological process are *P*-values (Fisher’s exact t-test), activation z-score, predicted activation state (based on a cut-off z-score = +/− 2), protein names and the number of proteins enriched in the corresponding cellular and biological process. Spreadsheet “Upstream regulators”: This table lists all the upstream regulators identified by IPA analysis for the SRMS-dependent upregulated/hyperphosphorylated proteins. Also shown are *p*-values, activation z-score, predicted activation state (based on a cut-off z-score = +/− 2), protein names and number of proteins for every upstream regulator identified by IPA analyses. Spreadsheet “Signaling pathways”: Shown here are the canonical signaling pathways corresponding to the upregulated/hyperphosphorylated proteins, as identified by IPA analysis. Also shown are the -log *p*-values, ratio (the ratio estimates the representation of the proteins in each canonical pathway. This is calculated as the ratio of the number of proteins that are enriched in the pathway to the number of proteins in the Ingenuity database reference dataset that make up the pathway) and the protein names corresponding to each canonical pathway. (XLSX 15 kb)

Additional file 4: **Figure S2.** A protein-protein interaction network map constructed using STRING [38] showing the intermolecular associations between the upregulated phosphoproteins (represented by nodes) at an interaction score threshold set to 0.4 (medium confidence). Nodes interconnected by an edge are representative of a protein-protein interaction context, as determined by experimental observations (edges highlighted in Pink), database searches (edges highlighted in blue) and text-mining (edges highlighted in green). (TIF 5774 kb)

Additional file 5: **Table S3.** This table lists all the candidate upstream kinases identified by NetworKIN analysis, for the upregulated phosphosites. Shown in the table is the substrate (phosphoprotein identified in the SRMS-regulated phosphoproteome), position (corresponding upregulated phosphosite), upstream kinase ID (candidate kinase known to target the corresponding phosphosite), NetworKIN score, kinase group (NetPhorest-annotated kinase group), NetPhorest score, STRING identifier (the corresponding STRING-annotated identifier/ID for the interaction between the kinase and its substrate phosphoprotein), STRING score, Motif sequence (the specific motif corresponding to the phosphosite that is targeted by the candidate kinase) and STRING path. (XLSX 16 kb)

## Abbreviations

BFK: BRK family kinase
BRK: Breast tumor kinase
Cdc2: Cell division cycle protein 2
CDK1: Cyclin-dependent kinase 1
CDK9: Cyclin-dependent kinase 9
CK2: Casein kinase 2
CK2α: Casein kinase 2 alpha
FDR: False discovery rate
FRK: Fyn-related kinase
HMGA1: High mobility group protein A1
*MYH9*: *Myosin heavy chain 9*
*NUCL*: *Nucleolin*
*PI3K*: *Phosphoinositol 3 kinase*
PTB: Phosphotyrosine-binding domain
PTK70: Protein tyrosine kinase 70
SFK: Src family kinases (SFKs)
SH2: Src-homology domain 2
SH3: Src-homology domain 3
SRMS: Src-related kinase lacking C-terminal regulatory tyrosine and N-terminal myristoylation sites
