# Supplementary figures and images for: Global phosphoproteomic analysis identifies SRMS-regulated secondary signaling intermediates
Source: Proteome Sci. 2018 Aug 18;16:16. doi: 10.1186/s12953-018-0143-7 (PMC6098843; doi:10.1186/s12953-018-0143-7)

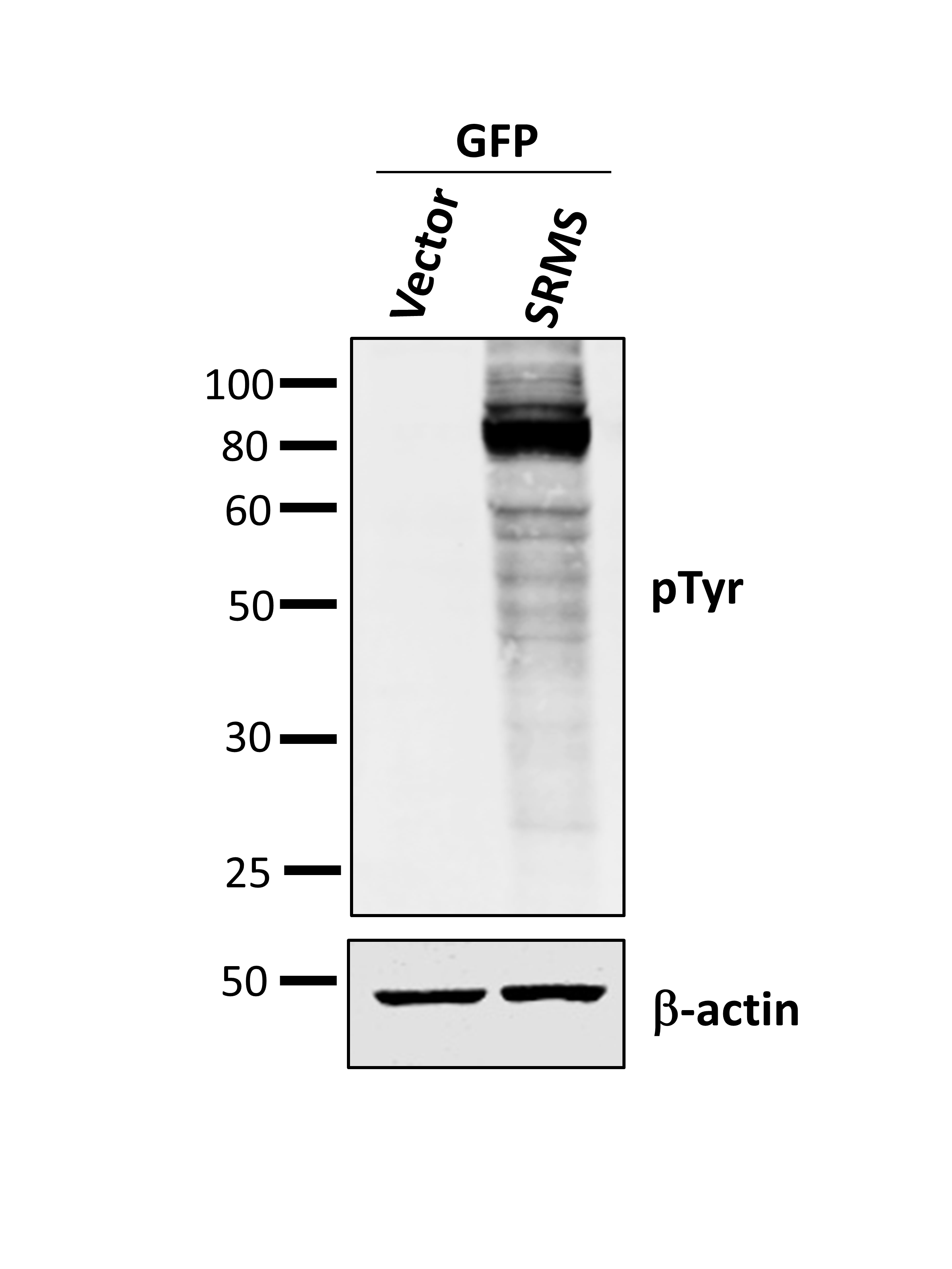

Supplement: Supplementary file 1 — Figure S1. Immunoblotting analysis with antibodies against total phosphotyrosines on lysates derived from cells expressing either empty vector control (GFP alone) or GFP-SRMS wild type. Antibodies against β-actin were used for immunoblotting to assess the loading of total proteins. (TIF 855 kb) [file 12953_2018_143_MOESM1_ESM.tif]

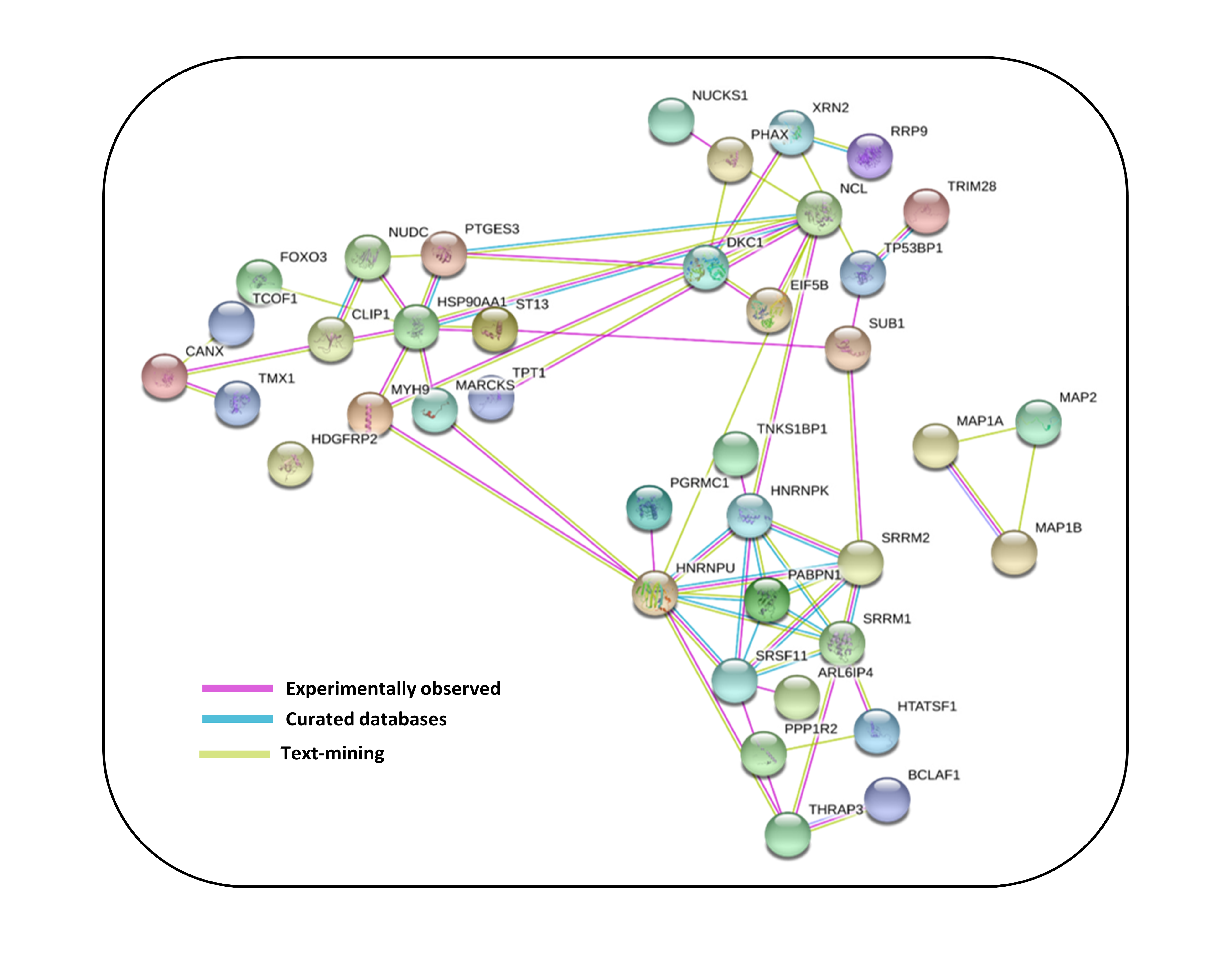

Supplement: Supplementary file 4 — Figure S2. A protein-protein interaction network map constructed using STRING [38] showing the intermolecular associations between the upregulated phosphoproteins (represented by nodes) at an interaction score threshold set to 0.4 (medium confidence). Nodes interconnected by an edge are representative of a protein-protein interaction context, as determined by experimental observations (edges highlighted in Pink), database searches (edges highlighted in blue) and text-mining (edges highlighted in green). (TIF 5774 kb) [file 12953_2018_143_MOESM4_ESM.tif]
